# The role of the Arabidopsis *FUSCA3 *transcription factor during inhibition of seed germination at high temperature

**DOI:** 10.1186/1471-2229-12-15

**Published:** 2012-01-27

**Authors:** Rex S Chiu, Hardeep Nahal, Nicholas J Provart, Sonia Gazzarrini

**Affiliations:** 1 Department of Biological Sciences, University of Toronto, 1265 Military Trail, Toronto, ON, M1C 1A4 Canada; 2 Department of Cell and Systems Biology, University of Toronto, 25 Harbord Street, Toronto, ON, M5S 3G5 Canada; 3 Centre for the Analysis of Genome Evolution and Function (CAGEF), University of Toronto, 25 Harbord Street, Toronto, ON, M5S 3G5 Canada

**Keywords:** High temperature, FUSCA3, Seed germination, Hormones, ABA, Transcriptome

## Abstract

**Background:**

Imbibed seeds integrate environmental and endogenous signals to break dormancy and initiate growth under optimal conditions. Seed maturation plays an important role in determining the survival of germinating seeds, for example one of the roles of dormancy is to stagger germination to prevent mass growth under suboptimal conditions. The B3-domain transcription factor FUSCA3 (FUS3) is a master regulator of seed development and an important node in hormonal interaction networks in *Arabidopsis thaliana*. Its function has been mainly characterized during embryonic development, where *FUS3 *is highly expressed to promote seed maturation and dormancy by regulating ABA/GA levels.

**Results:**

In this study, we present evidence for a role of *FUS3 *in delaying seed germination at supraoptimal temperatures that would be lethal for the developing seedlings. During seed imbibition at supraoptimal temperature, the *FUS3 *promoter is reactivated and induces *de novo *synthesis of *FUS3 *mRNA, followed by FUS3 protein accumulation. Genetic analysis shows that *FUS3 *contributes to the delay of seed germination at high temperature. Unlike WT, seeds overexpressing *FUS3 *(*ML1:FUS3-GFP*) during imbibition are hypersensitive to high temperature and do not germinate, however, they can fully germinate after recovery at control temperature reaching 90% seedling survival. *ML1:FUS3-GFP *hypersensitivity to high temperature can be partly recovered in the presence of fluridone, an inhibitor of ABA biosynthesis, suggesting this hypersensitivity is due in part to higher ABA level in this mutant. Transcriptomic analysis shows that WT seeds imbibed at supraoptimal temperature activate seed-specific genes and ABA biosynthetic and signaling genes, while inhibiting genes that promote germination and growth, such as GA biosynthetic and signaling genes.

**Conclusion:**

In this study, we have uncovered a novel function for the master regulator of seed maturation, *FUS3*, in delaying germination at supraoptimal temperature. Physiologically, this is important since delaying germination has a protective role at high temperature. Transcriptomic analysis of seeds imbibed at supraoptimal temperature reveal that a complex program is in place, which involves not only the regulation of heat and dehydration response genes to adjust cellular functions, but also the activation of seed-specific programs and the inhibition of germination-promoting programs to delay germination.

## Background

Seed maturation begins during mid-embryogenesis and controls important traits, including seed quality and viability. In Arabidopsis, during mid-embryogenesis the embryo accumulates storage compounds, such as seed storage proteins (SSP) and lipids, which are broken down during the first days of germination to sustain the growing seedling. As development progresses into late embryogenesis a variety of protective proteins, including late embryogenesis abundant proteins (LEA) and heat shock proteins (HSP), are synthesized to prepare the embryo for desiccation and the final stage of dormancy [1]. Genetic and molecular studies have shown that seed maturation is orchestrated by the hormone abscisic acid (ABA) and a network of transcription factors, which include the *LEAFY COTYLEDON1 *(*LEC1*) CCAAT binding factor and the B3-domain transcription factors, *LEC2*, *FUSCA3 *(*FUS3*) and *ABSCISIC ACID INSENSITIVE3 *(*ABI3*) [2-4]. These genes are considered global regulators of seed maturation, as loss-of-function mutant embryos skip late-embryonic development and enter the vegetative program prematurely. As a consequence, immature mutant seeds are less dormant, can germinate precociously and accumulate less seed storage compounds. Furthermore, seeds of *fus3*, *abi3 *and *lec1 *mutants are also desiccation intolerant and become inviable when dry [5-11]. Enhancement of these phenotypes shown in higher order mutants, and in transcriptional regulatory studies, demonstrates that these genes act in concert to control seed maturation, dormancy and desiccation tolerance [8-10,12-14]. The important role of ABA in regulating seed maturation and dormancy is exemplified by genetic and chemical studies, which show that a reduction of ABA level also decreases seed dormancy [3,15]. Further studies have shown that B3-domain transcription factors are important nodes in hormone signaling pathways and control important steps in hormone biosynthesis. For example, *FUS3 *promotes dormancy and prevents precocious germination of immature seeds, by stimulating ABA synthesis while repressing GA biosynthesis [16-18]. In a feedback regulation, ABA and GA respectively stabilize and destabilize the FUS3 protein through a mechanism involving the C-terminal region of FUS3 [17,19]. In addition to its role in seed development, *ABI3 *is a positive regulator of ABA signaling, as *abi3 *seeds are strongly insensitive to ABA during germination [5,6,20]. ABA sensitivity is not affected in *fus3, lec1 *and *lec2*, suggesting that ABA sensitivity and seed maturation can be uncoupled [9,10]. The B3-domain proteins are also involved in auxin sensitivity and biosynthesis. The promoters of *FUS3 *and *ABI3 *are induced by auxin, while auxin biosynthetic genes are positively regulated by *LEC2 *and *FUS3 *[17,21-23].

The B3-domain binds the Sph/RY (CATGCA) core element found in the promoter of many seed-specific genes and some of the B3-domain targets include *LEA *and *Em *[24-26]. These genes are also under the control of ABA [27]. RY/Sph elements have been shown to act synergistically with ABA response elements (ABRE), suggesting that a complex interaction of seed-specific and ABA-regulated factors controls late embryogenesis and seed maturation [28-30]. *Heat shock factors *(*HSF*) and *HSPs*, which are rapidly induced during heat stress (HS) and highly abundant in seeds, are also B3-domain and ABA targets. In particular, the expression of the seed specific *HSFA9 *is induced by *ABI3 *through the Sph/RY in an ABA-dependent manner. Activation of *HSFA9*, in turn, regulates the expression of select *HS *genes [31].

Transcript levels of the *LEC, FUS3 *and *ABI3 *genes are abundant during embryogenesis, decrease during germination, and are expressed at low levels in vegetative organs [13,19,32-34]. Repression of these genes during germination is required to enter vegetative development, as overexpression of *FUS3 *and the *LEC *post-embryonically results in the expression of embryonic markers and the development of embryonic structures during vegetative growth [17,33,35-37]. More specifically, overexpression of *FUS3 *(*ML1:FUS3-GFP*) results in delayed seed germination and hypersensitivity to ABA. Since one of the roles of dormancy is to stagger germination and prevent mass growth under suboptimal conditions, we tested whether during imbibition under abiotic stress *FUS3 *would be re-activated to delay germination. Among the stresses tested in this study, we found that high temperature induces *de novo FUS3 *mRNA synthesis and protein accumulation in imbibed seeds. In contrast to WT, *ML1:FUS3-GFP *seeds overexpressing *FUS3 *during germination are hypersensitive to supraoptimal (high) temperature and fail to germinate. Inhibition of *ML1:FUS3-GFP *germination at supraoptimal temperature is partly dependent on ABA biosynthesis and is a reversible process, as *ML1:FUS3-GFP *seeds imbibed at high temperature for six days can resume germination when the temperature becomes optimal for growth. Transcriptomic analysis shows that seeds exposed to supraoptimal temperature re-activate the expression of late embryonic and seed maturation programs, while inhibiting germination-promoting programs, to delay seed germination at supraoptimal temperatures.

## Results

### Germination at supraoptimal temperature activates the *FUS3* promoter and induces FUS3 mRNA and protein accumulation

**Figure 1:**
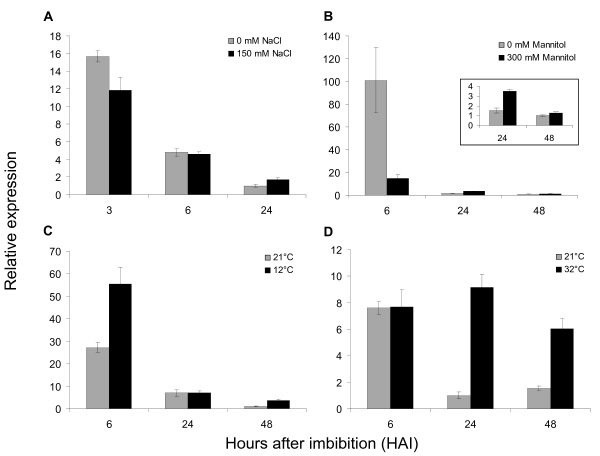
**Transcriptional regulation of *FUS3 *by abiotic stresses**. (A-D) Relative *FUS3 *mRNA levels measured by qPCR in mature seeds exposed to abiotic stresses during imbibition under constant light. A) salt, 150 mM NaCl; B) osmotic, 300 mM mannitol; C) low temperature, 12°C and D) high temperature, 32°C. The mean value of three replicates was normalized using *ACTIN 7 *as the internal control. Results are plotted as the ratio to the lowest detected level. Two independent experiments were conducted with similar results and one representative is shown.

To test whether *FUS3 *plays a role during germination under abiotic stresses, we measured *FUS3 *mRNA levels in WT seeds imbibed on high salt (150 mM NaCl), during osmotic stress (300 mM mannitol) and at low (12°C) and high (32°C) temperatures (Figure 1). These conditions have been previously shown to delay WT seed germination [38]. We screened for changes in patterns of *FUS3 *expression at early (3 or 6 h) and late (24 or 48 h) stages of seed imbibition, since *FUS3 *transcript level rapidly decreases during germination [19]. *FUS3 *mRNA level is only slightly affected on 150 mM NaCl and fluctuates in expression on 300 mM mannitol and at 12°C. In contrast, *FUS3 *mRNA level increases 2-4 fold in seeds imbibed at 32°C for 24 h and 48 h, therefore, we investigated the role of *FUS3 *during seed germination at high temperature in more detail.

**Figure 2:**
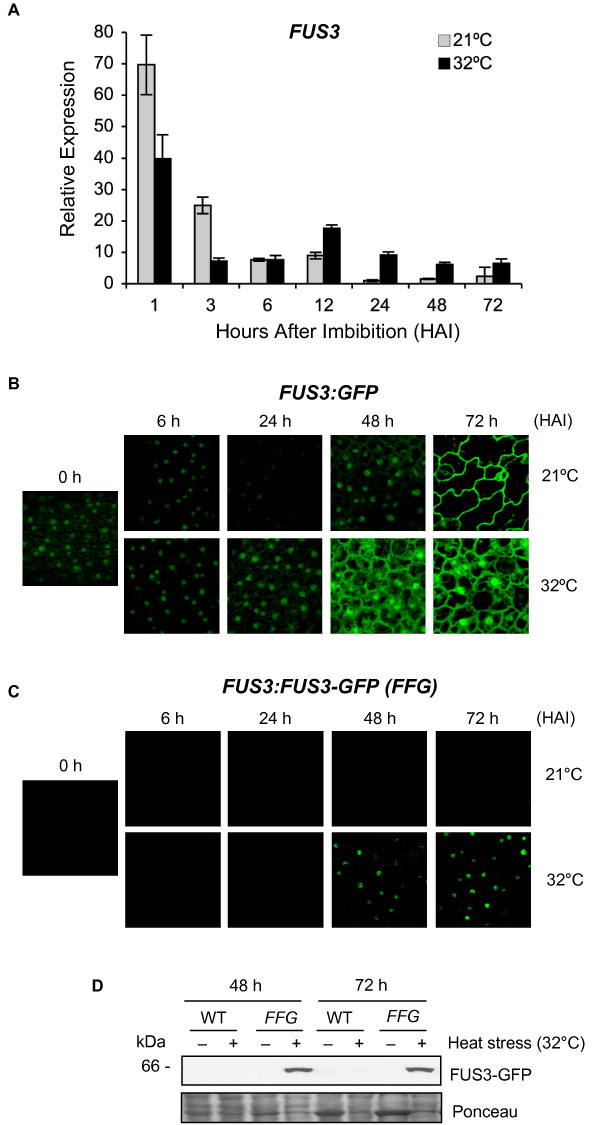
**Seed imbibition at supraoptimal temperature activates the *FUS3 *promoter and induces de novo FUS3 mRNA synthesis and FUS3 protein accumulation**. (A) Relative *FUS3 *mRNA levels measured by qPCR in seeds imbibed at 21°C or 32°C for up to 72 h under constant light. The mean value of three replicates was normalized using *ACTIN 7 *as the internal control. Results are plotted as the ratio to the lowest detected level. Two independent experiments were conducted with similar results and one representative is shown. (B) Confocal images showing GFP fluorescence in the epidermis of *FUS3:GFP *embryos of seeds imbibed at 21°C or 32°C. HAI, hours after imbibition. (C, D) Confocal images (C) and immunoblots (D) showing FUS3-GFP fluorescence and protein accumulation in *FUS3p:FUS3-GFP (FFG) *seeds imbibed at 21°C and 32°C for up to 72 h under constant light. WT in D) is shown as the negative control. FUS3-GFP (~MW 61 kDa) was detected with anti-GFP antibody. Comparable confocal settings were used in all images shown in B) and C). Duplicate experiments were conducted and one representative is shown.

We performed a more detailed time-course experiment and monitored *FUS3 *expression level in seeds imbibed at 32°C for up to 3 days (Figure 2A). We found that the level of *FUS3 *mRNA decreases faster during the first 3 h of HS, reaching a comparable level to the control at 6 h. Thereafter, the mRNA starts increasing and remains higher than the control for at least 3 days (Figure 2A). To test whether the increase in *FUS3 *mRNA after longer HS exposure was due to an activation of the *FUS3 *promoter or an increase in *FUS3 *mRNA stability, we used the *FUS3:GFP *transcriptional reporter previously described (Figure 2B) [19]. Compared to the 21°C control, there is a marked increase in GFP fluorescence at 32°C between 24 and 72 h, which reflects an increase in *FUS3 *promoter activity. Thus, the exposure of seeds to high temperatures induces rapid degradation of *FUS3 *mRNA within the first 3 h, followed by transcriptional activation (or derepression) leading to new mRNA synthesis from 12 h. These findings are mirrored by an increase in FUS3 protein level, measured by using the *FUS3:FUS3-GFP *(*FFG*) translational reporter previously described (Figure 2C, D) [17]. The FUS3-GFP protein was detected in the nuclei of epidermal cells and on immunoblots of seeds imbibed at 32°C for 48 and 72 h (Figure 2C, D). In agreement with previous studies, no FUS3-GFP was detected in seeds imbibed at 21°C for up to 3 days (Figure 2C, D) [19].

Next, we tested whether the late accumulation of the FUS3-GFP protein at 32°C was due to an increase in the seed ABA/GA ratio, as Arabidopsis seeds imbibed at high temperature repress GA biosynthesis and induce *de novo *ABA synthesis resulting in a higher ABA level at 24 h [39]. No FUS3-GFP was detected in *FUS3:FUS3-GFP *seeds germinated on ABA or paclobutrazol, a GA biosynthesis inhibitor, at 21°C, suggesting that FUS3 accumulation at high temperature is not dependent solely on higher ABA level (Additional file 1). Interestingly, the FUS3 protein was detected only in seeds that have commenced germination (radicle protrusion), but not in thermoinhibited seeds (no radicle protrusion) or seedlings that have germinated from the seed coat. This suggests that during germination at high temperature, FUS3 may act in a small developmental window that precedes seedling emergence from the seed coat. Together, these results indicate that imbibition at the supraoptimal temperature of 32°C induces an increase in *FUS3 *transcript level at 12-24 h, which is followed by an increase in FUS3 protein level at 48 h.

### Seed germination and seedling survival of WT and *fus3 *loss- and gain-of-function mutants imbibed at high temperature

**Figure 3:**
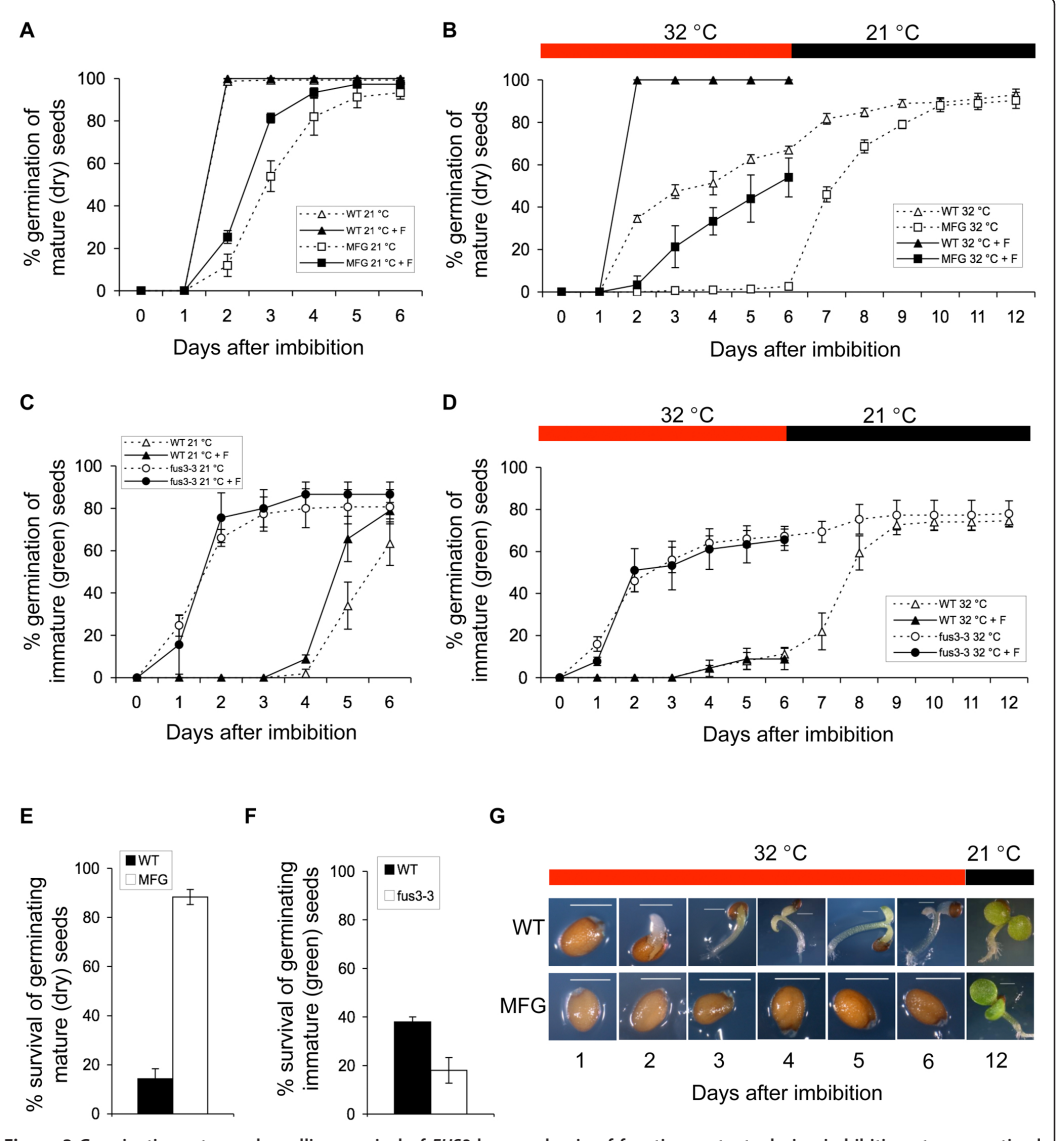
**Germination rates and seedling survival of *FUS3 *loss- and gain-of-function mutants during imbibition at supraoptimal temperature and recovery after heat stress**. (A-G) Germination rates and% of seedlings survival of wild type (WT), *FUS3 *loss-of-function mutant (*fus3-3*) and *ML1:FUS3-GFP *(*MFG*) gain-of-function mutant during imbibition at control (21°C) and supraoptimal temperature (32°C). (A, B, E) Germination (radicle protrusion) rates of mature WT and *MFG *dry seeds imbibed on filter papers on MS plates in the absence or presence of 10 μM fluridone (+F) at 21°C (A) and 32°C (B). After six days of imbibition at 32°C, plates in B) were transferred to the control temperature (21°C) for six additional days and the% of seedlings survival (showing green cotyledon and green leaves) was calculated in (E). (C, D, F) Germination (radicle protrusion) rates of immature WT and *fus3-3 *green seeds imbibed on filter papers on MS media in the presence or absence of 10 μM fluridone (+F) at 21°C (C) and 32°C (D). After six days of imbibition at 32°C, plates in D) were transferred to the control temperature (21°C) for six additional days and the% of seedlings survival (showing green cotyledon and green leaves) was calculated in (F). (G) Appearance of representative WT and MFG seeds from the experiment shown in B). Averages from triplicates ± s.d. are shown (n ≥ 300 seeds). Experiments were repeated at least twice with similar results and one representative is shown. F, fluridone. Red bars, 32°C. Black bars, 21°C.

To dissect the role of *FUS3 *during germination at supraoptimal temperatures, we monitored the germination and survival rates of WT, *fus3 *loss-of-function (*fus3-3*) and overexpression (*fus3-3*, *ML1:FUS3-GFP*) mutant seeds imbibed at 32°C (Figure 3). The *ML1:FUS3-GFP *construct was previously shown to rescue the *fus3-3 *loss-of-function mutant and overexpress *FUS3 *post-embryonically throughout vegetative and reproductive development [17]. At 21°C, mature WT seeds reach 100% germination within 2 days, while *ML1:FUS3-GFP *seeds are more dormant and germinate slower reaching 90% germination in 5-6 days (Figure 3A). Despite reaching 60-100% germination within 6 days (depending on seed batches), mature WT seeds show delayed germination at 32°C (Figure 3B). All grown seedlings manifest phenotypes associated with high temperature stress, including arrested growth and bleached cotyledons and hypocotyls (Figure 3G). These seedlings do not recover and fail to grow even when transferred to 21°C, indicating that prolonged exposure to 32°C is lethal for Arabidopsis seedling growth (Figure 3B, E). In contrast, germination of mature *ML1:FUS3-GFP *seeds was greatly reduced or inhibited (depending on seed batches) for up to 6 days at 32°C, suggesting *ML1:FUS3-GFP *seeds are hypersensitive to high temperatures (Figure 3A, B, G). Although a very low percentage of seeds show radicle protrusion between 3 and 6 days, no seedling emerges from the seed coat (Figure 3B, G). Interestingly, all *ML1:FUS3-GFP *seeds resumed germination when transferred to the control temperature and this results in a much greater *ML1:FUS3-GFP *survival rate compared to the WT (Figure 3B, E).

Mature *fus3 *loss-of-function (*fus3-3*) mutant seeds are desiccation intolerant and inviable, but can be rescued if immature seeds are harvested when still green, prior to desiccation. Therefore, we compared the germination rates of immature green seeds of *fus3-3 *and WT when imbibed under control and high temperature (Figure 3C, D). At 21°C, immature WT seeds begin to germinate 4 days after imbibition and reach ~60% germination by 6 days. This is expected, as immature WT seeds are dormant. However, at 32°C, immature WT seeds show strong thermoinhibition and reach ~10% germination after 6 days. After transfer to 21°C, WT immature seeds resume germination. In contrast, immature *fus3-3 *seeds reach ~80% and ~60% germination by 6 days at 21°C and 32°C, respectively, and thus are strongly resistant to thermoinhibition. As expected, immature *fus3-3 *seeds, which have a higher percentage of germination than immature WT seeds at 32°C, showed lower seedling survival than WT after recovery at 21°C (Figure 3D, F). In summary, this data indicates that overexpression of FUS3 inhibits seed germination at supraoptimal temperature and this increases seedling survival when temperatures become optimal for growth. It also suggests that inhibition of germination at supraoptimal temperature is a reversible process and has a protective role for the embryo.

### *ML1:FUS3-GFP* hypersensitivity to high temperature during imbibition is dependent on ABA biosynthesis

Arabidopsis seeds imbibed at 34°C have higher ABA/GA ratio due to the activation of ABA and repression of GA biosyntheses, which results in thermoinhibition of seed germination [39]. *ML1:FUS3-GFP *seeds are hypersensitive to ABA during germination and transient activation of FUS3 using an *ML1:FUS3-GR *construct increases ABA levels post-embryonically [17]. In light of this, we investigated whether *ML1:FUS3-GFP *inhibition of germination at 32°C is due to altered ABA biosynthesis. To test this, we germinated WT and *ML1:FUS3-GFP *seeds in the presence of fluridone, an inhibitor of ABA biosynthesis. Fluridone can fully reverse the delay of WT germination at 32°C and partly recover the inhibition of *ML1:FUS3-GFP *germination at 32°C (Figure 3B). Fluridone also increases the germination rate of *ML1:FUS3-GFP *seeds at 21°C (Figure 3A). This suggests that the delayed germination of *ML1:FUS3-GFP *seeds at the control temperature, and inhibition at high temperature, appear to be partly dependent on *de novo *ABA biosynthesis. Furthermore, since seeds overexpressing FUS3 are more dormant, partly due to increased ABA biosynthesis, increased dormancy may have a positive effect during germination at supraoptimal temperatures.

In contrast, fluridone did not have any effect on the germination of immature *fus3-3 *and WT seeds at 32°C, and slightly increased the germination of WT seeds at 21°C (Figure 3C). The insensitivity of *fus3-3 *seeds to fluridone was expected, as *fus3-3 *seeds have a lower ABA level [16,17]. However, the lack of effect of fluridone on the germination of immature WT seeds was surprising. Since immature WT seeds resume germination when plates are shifted to the optimal temperature, the lack of effect of fluridone on WT germination cannot be attributed to damage or death of the WT green seeds. This suggests that *de novo *ABA synthesis may not have a predominant role in regulating the germination of WT immature seeds at high temperature (Figure 3D and 3E).

### Transcriptomic analysis of seeds imbibed at high temperature

Our data shows that seed germination at the supraoptimal temperature of 32°C induces *de novo FUS3 *mRNA synthesis and, most importantly, FUS3 protein accumulation. Furthermore, *FUS3 *overexpression strongly inhibits Arabidopsis germination partly through ABA biosynthesis. Since high temperature induces not only changes in ABA/GA levels but also reactivates *FUS3 *function in imbibed seeds, we predicted that high temperature would also activate seed maturation programs partly regulated by the B3-protein network to delay germination. Furthermore, since FUS3 controls the ABA/GA ratio and since ABA and GA have been shown to play important roles during thermoinhibition, we were interested in identifying hormonal networks working at the time FUS3 is active. Therefore, we characterized the transcriptomic response of WT seeds imbibed at high temperature. We conducted a time-course microarray (Affymetrix ATH1) with WT Arabidopsis seeds imbibed at optimal (21°C) or supraoptimal (32°C) temperatures for 1, 12 and 24 h. These time points were chosen to identify early (1 h) and late (12 and 24 h) heat-stress responsive (*HSR*) genes. The latter time points were also chosen because they correlate with an increase in ABA level and *FUS3 *expression, and thus may identify *FUS3 *and ABA coexpressed genes.

**Figure 4:**
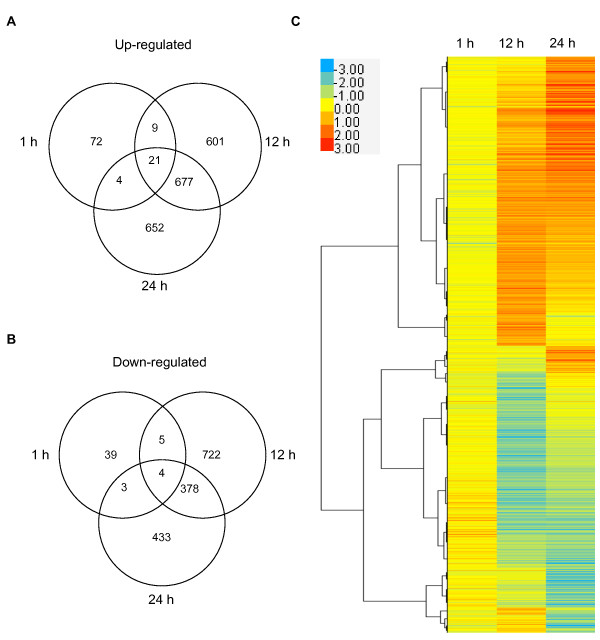
**Cluster analysis of differentially expressed *HSR *genes**. (A, B) Venn diagrams showing the number of transcripts with enhanced (A) or decreased (B) expression levels in seeds imbibed for 1, 12 and 24 h at 32°C compared to 21°C. Only genes showing a minimum fold change 1.8 (log_2 _≥ 0.85) and a *p*-value < 0.05 were selected. Results are presented as averages of two independent experiments. (C) Heat map of differentially expressed genes in seeds imbibed for 1, 12 and 24 h at 32°C compared to 21°C based on hierarchical clustering. Red color indicates upregulated genes, blue color indicates downregulated genes and yellow color indicates genes whose expression is unchanged.

Only a small set of 157 genes change in expression level after 1 h of heat treatment, while > 2000 genes are differentially expressed at both 12 and 24 h, indicating a large transcriptional reprogramming at later stages of HS (Figure 4; Additional files 2, 3, 4). While there is a relatively small overlap between early and late *HSR *genes, a large number of genes show increased (677) or decreased (378) expression at both 12 and 24 h. To determine the cellular responses to HS, we analyzed transcriptional changes using the Gene Ontology (GO) enrichment analysis (Additional files 5, 6, 7, 8, 9, 10) [40]. At all time points, the largest GO-function categories being enriched are those comprising 'cellular and metabolic processes', while the most significant function being enriched is the 'response to stimulus' (Additional file 5). After 1 h of exposure to 32°C the imbibed seed has limited response, and genes important for the 'response to high temperature' and 'protein folding' are among the most enriched in early *HSR*s (Additional file 6). After 12 and 24 h of imbibition at 32°C, however, the cellular response of the seed becomes more complex. GO-enrichment analysis suggests that the specificity of the HS response is strengthened by activating genes required for thermotolerance, adjusting cellular metabolism and inhibiting growth and development (Additional files 7, 8, 9, 10). While the most significant biological function enriched in late *HSR*s is again the 'response to stimulus', 'embryonic development' and 'seed dormancy' are also among the GO-function categories significantly enriched (Additional files 7, 8, 9, 10). The most enriched 'stimuli' include abiotic (high temperature, oxidative, high light and water deprivation) and chemical (ABA, GA and auxin) stimuli.

### Stress-related and seed maturation genes are activated during seed imbibition at supraoptimal temperature

**Figure 5:**
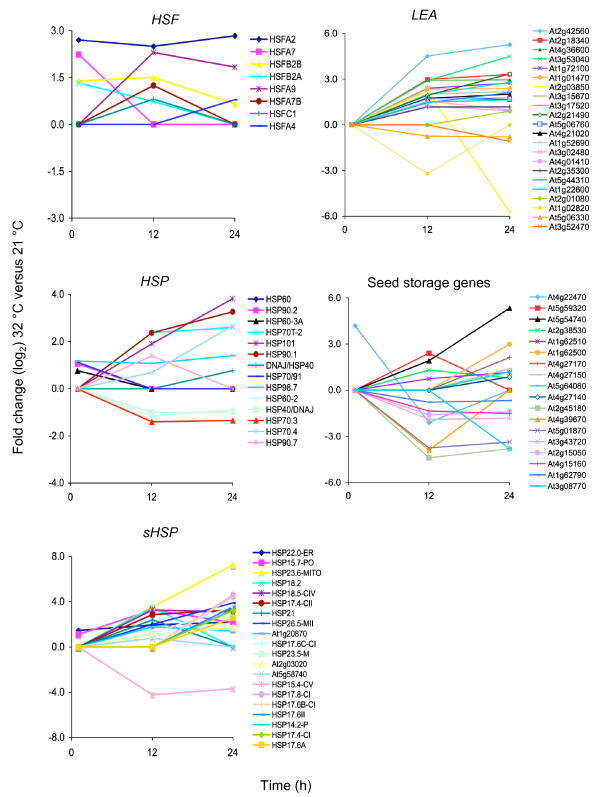
**Time-course expression analysis of HS-related and seed maturation genes**. Expression patterns of select genes involved in heat-shock response (*Heat Shock Protein, HSP; *small *HSP, sHSP; Heat Shock Factors, HSF*) and late-embryogenesis abundant (*LEA*) and seed maturation (storage lipids and proteins) genes after 1, 12 and 24 h of imbibition at 32°C compared to 21°C. Y axis, fold-changes (log_2_) in genes expression levels at 32°C compared to 21°C. × axis, hours (h) after imbibitions.

*HSPs *and *HSFs *are well known for their protective role in cellular functions at high-temperature and are rapidly induced after HS, however, they also play a role in other stresses [41]. Seven *HSPs *and four *HSFs *increase in expression at 1 h, including *HSFA2 *and *HSFA7*, which have been well characterized for their role in the acquisition of thermotolerance in seedlings (Figure 5) [42,43]. A larger number of *HSPs *(49 genes) and *HSFs *(7 genes) show an increase in transcript abundance at 12 h and/or 24 h, with a strong induction of the highly expressed *HSFA2 *and the proposed seed-specific and HS-independent, *HSFA9 *(Figure 5) [31]. These late-induced *HSF *genes may be required for prolonged cellular responses to high temperature.

As expected, seeds imbibed at high temperature for 12 and 24 h show a large increase in the expression levels of late embryogenesis and seed maturation genes, including *LEA, SSPs *and storage lipids (42 genes; Figure 5). Together with molecular chaperones and other protective compounds, *LEA *genes are thought to be involved in direct abiotic stress tolerance and are induced by several abiotic stresses [41]. SSP and storage lipids are typical markers of seed maturation and some have been shown to be regulated directly or indirectly by B3-domain proteins, including FUS3 [6-10,13,23-26,28,37]. Among the known global regulators of late embryogenesis and seed maturation, *FUS3 *is the only gene induced by HS, suggesting that it may play an important role in the activation of seed-specific programs during imbibition at supraoptimal temperature (Additional file 4, Additional file 5). Interestingly, transcript levels of three yet uncharacterized B3-containing proteins (At4g33280, At2g35310, At5g25475) are also induced at 24 h (Additional file 5), and may play complementary roles with FUS3 during imbibition at supraoptimal temperature.

### Prolonged HS activates ABA and represses GA metabolic genes

**Figure 6:**
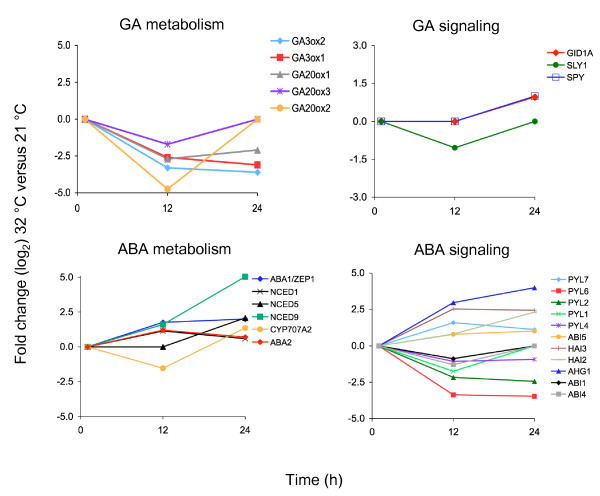
**Time-course expression analysis of select *HSR *genes**. Expression patterns of hormone (ABA and GA) metabolism and signaling genes after 1, 12 and 24 h of imbibition at 32°C compared to 21°C. Y axis, fold-changes (log_2_) in genes expression levels at 32°C compared to 21°C. × axis, hours (h) after imbibitions.

Genes involved in hormone metabolism and signaling, predominantly the hormones ABA and GA, are enriched among the late *HSR *genes (Additional file 7, 8, 9, 10). Genes regulating ABA metabolic pathways show increased mRNA levels, while transcript levels of GA biosynthesis genes are decreased (Figure 6). Only a few known GA signaling genes were identified as *HSR *in our microarray, including the GA receptor, *GID1A*, and signaling components such as *SPY *and *SLY*. In contrast, several genes involved in ABA signaling are differentially expressed at high temperature. These include several members of the ABA receptor family *(PYL/RCARs*), as well as other downstream signaling components such as PP2C phosphatases (*ABI1*, *HAI2*, *HAI3*, *AHG1*) and transcription factors (*ABI4*, *ABI5*) (Figure 6). More than 100 kinases have altered expression level during heat stress, including members of the *SnRK2/3 *families of serine/threonine kinases, which comprises positive regulators of ABA signaling and abiotic stress responses (Additional file 3, Additional file 4) [44] . Most of these genes, including several *PYL/RCARs*, *PP2Cs *and *SnRK2/3 *kinases have uncharacterized functions. Thus, transcriptomic analysis of seeds imbibed at high temperature suggests that select known ABA signaling genes as well as uncharacterized members of the *PYL/RCAR*, *PP2C *and *SnRK *families are differentially regulated during germination at supraoptimal temperature and may play specific roles during thermoinhibition.

A number of genes coding for different families of transcription factors involved in other hormone or stress signaling pathways are also differentially regulated at 12 and 24 h. Transcript levels of the drought and heat-stress responsive *DREB2A *[45], auxin (*ARF11*, *ARF18 *and several *SAUR*) and ethylene (*EIN3, EIN4, ERSs*, *ERFs*) responsive genes are among some of those that fluctuate in expression level at 12 and/or 24 h (Additional file 3, Additional file 4). Thus, other hormones beside ABA and GA may play a role during germination at supraoptimal temperature.

### Core *HSR *genes

The transcript levels of 25 genes are increased or decreased at all three time points and constitute the core *HSR *genes (Table 1). Beside the constitutively and highly expressed *HSFA2*, all other genes in this list have not been tested for their role during HS. Interestingly, *PDX1.2 *is highly expressed and is among the strongest *HSR *genes induced at 1 h. Mutations in *PDX1 *genes affect pyridoxine (vitamin B6) biosynthesis, however, the role of *PDX1.2 *has not been clarified yet [46]. Vitamin B6 is known to play a protective role against oxidative stress and it is a cofactor in many biochemical processes [46,47]. Thus, vitamin B6 may be an important component in the response of seeds to high temperature.

**Table 1:** Core *HSR *genes.

*A) Core HSR genes upregulated at 1, 12 and 24 h*	
**AGI**	**Annotation**

At3g29810	COBRA-like protein 2 precursor (COBL2)

At5g56600	profilin 3 (PFN3_PRF3)

At5g48570	FKBP-type peptidyl-prolyl cis-trans isomerase (FKBP65_ROF2)

At5g48480	Lactoylglutathione lyase/glyoxalase I

At5g37670	HSP20-like chaperones

At5g12110	Glutathione S-transferase, C-terminal-like; Translation elongation factor EF1B/ribosomal protein S6

At5g09590	mitochondrial HSO70 2 (HSC70-5_MTHSC70-2)

At4g29770	Target of trans acting-siR480/255

At4g23570	SGT1A_phosphatase-related

At4g02980	endoplasmic reticulum auxin binding protein 1 (ABP1)

At4g10250	ATHSP22.0_HSP20-like chaperones

At1g66080	unknown protein

At3g14880	unknown protein

At3g24500	multiprotein bridging factor 1 C (ATMBF1C)

At3g16050	pyridoxine biosynthesis 1.2 (A37_ATPDX1.2)

At3g07150	unknown protein

At1g07350	RNA-binding protein (RRM/RBD/RNP motifs)

At1g26800	RING/U-box superfamily protein

At1g35660	unknown protein

At2g25140	casein lytic proteinase B4 (CLPB-M_CLPB4_HSP98.7)

At2g26150	heat shock transcription factor A2 (ATHSFA2)

***B) Core HSR genes downregulated at 1, 12 and 24 h***	

**AGI**	**Annotation**

At5g51190	Integrase-type DNA-binding

At3g48140	B12D protein

At3g08730	protein-serine kinase 1 (ATPK1_ATPK6_ATS6K1)

At1g28110	serine carboxypeptidase-like 45 (SCPL45)

List of *HSR *genes whose expression level is increased or decreased in seeds imbibed at 32°C at all time points (1, 12 and 24 h). Genes were filtered as described in the methods

## Discussion

**Figure 7:**
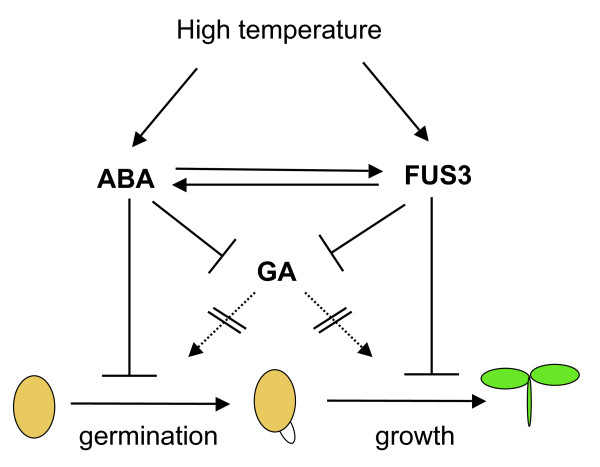
**Proposed model of thermoinhibition of seed germination**. During seed germination at supraoptimal temperature, induction of ABA and repression of GA syntheses and signaling delays germination. FUS3 acts in a short developmental window to prevent seedling growth, by positively regulating ABA while negatively regulating GA biosyntheses. ABA increases FUS3 stability by positive feedback regulation.

Seed germination is controlled by endogenous factors, such as the degree of dormancy, as well as environmental cues, including light, temperature and water. In Arabidopsis, germination of WT seeds is delayed or moderately inhibited at temperatures between 28 and 32°C, but completely inhibited at 34°C [39,48]. Physiological, genetic and molecular studies have highlighted the important and opposite roles of ABA and GA in promoting and alleviating thermoinhibition of seed germination, respectively [39,48-52]. The master regulator of seed maturation, *FUS3*, controls the ABA/GA ratio by negatively regulating GA biosynthesis, while positively regulating ABA levels [16,17]. Here, we show that one of the functions of *FUS3 *during post-embryonic development is to regulate seed responses to high temperatures, by delaying seed germination and inhibiting seedling growth partly through the regulation of ABA biosynthesis (Figure 7; see also below).

### High temperature induces *de novo FUS3 *mRNA synthesis and FUS3 protein accumulation during imbibition

*FUS3 *plays a predominant role during mid-late embryogenesis by promoting seed dormancy and maturation, while inhibiting precocious germination of immature seeds [10]. Accordingly, *FUS3 *mRNA level is high during mid-late embryogenesis and dramatically decreases during germination and throughout vegetative growth [19,32]. Furthermore, the level of the FUS3 protein does not correlate with that of its mRNA and remains undetected after mid-embryogenesis [17,19]. Given the very low level of *FUS3 *expression post-embryonically, one question raised by these studies is the physiological role of *FUS3 *during post-embryonic development. Here we found that seeds imbibed at 32°C rapidly degrade *FUS3 *mRNA stored in the seed and induce *de novo FUS3 *mRNA synthesis at 12 h, which is then followed by the accumulation of the FUS3 protein by 48 h (Figure 2). Notably, the *FUS3 *mRNA levels at 32°C are ~15 to 70-fold lower than those measured 1 h after imbibition at 21°C. Despite the lower mRNA level, the FUS3 protein is detected in seeds imbibed at 32°C but not at 21°C. We have previously shown that FUS3 is a short-lived protein and posttranslational regulation is one of the mechanisms that maintains FUS3 protein levels very low (undetectable) after mid-embryogenesis and during germination [19]. Therefore, it is likely that the accumulation of the FUS3-GFP protein at 32°C is due to translational and/or posttranslational regulation induced by HS.

Considering the FUS3-GFP protein is not detected in *FUS3:FUS3-GFP *seeds imbibed on ABA at 21°C (Additional file 1), ABA alone does not seem to be sufficient to induce FUS3-GFP protein accumulation during imbibition at high temperature. However, ABA may be required to maintain FUS3 protein levels high by a positive feedback regulation, as ABA was previously shown to positively regulate FUS3 abundance in *ML1:FUS3-GFP *seedlings (Figure 7) [17]. Interestingly, we found that at 32°C the FUS3 protein can only be detected in seeds that have commenced germination (radicle protrusion from the seed coat) and is not detected in inhibited seeds (no radicle emergence) or in seedlings that have fully emerged from the seed coat. This indicates that FUS3 is only active in a small developmental window during germination at high temperature and may be important to delay germination and inhibit seedling growth (Figure 7; see also below).

### Overexpression of *FUS3 *inhibits seed germination at supraoptimal temperature partly through positive regulation of ABA biosynthesis

Overexpression of FUS3 has a protective role for the embryo during germination at high temperature. Indeed, at 32°C WT seeds show delayed germination compared to the optimal temperature of 21°C and suffer from heat-stress related damages. In contrast, seeds overexpressing *FUS3 *post-embryonically (*ML1:FUS3-GFP*) show a strong thermoinhibition and result in a much higher seedlings survival than WT when shifted at 21°C (Figure 3). Thermoinhibition exhibited by *ML1:FUS3-GFP *seeds at 32°C can be alleviated by the ABA biosynthesis inhibitor, fluridone, suggesting it is dependent on *de novo *ABA biosynthesis. Furthermore, *ML1:FUS3-GFP *seeds are more dormant than WT at 21°C and the delayed germination exhibited by *ML1:FUS3-GFP *seeds at optimal temperature can also be rescued by fluridone. Considering that transient activation of FUS3 post-embryonically increases ABA levels and that *ML1:FUS3-GFP *seeds are hypersensitive to ABA even at the optimal temperature of 21°C [17], we conclude that seeds overexpressing *FUS3 *are hypersensitive to high temperature and induces thermoinhibition through *de novo *activation of ABA biosynthesis. These findings mirror previous findings showing that thermoinhibition of germination at 34°C is partly due to *de novo *ABA biosynthesis and that loss-of-function mutants affected in ABA biosynthesis show resistance to thermoinhibition [39]. Thus, increased seed dormancy and/or ABA biosynthesis is advantageous as it delays or prevents germination at high temperatures and protects the developing seedling from temperature-induced damage.

Immature green seeds of the *fus3-3 *loss-of-function mutant, which accumulate less ABA during embryogenesis, are less dormant than WT at 21°C (Figure 3) [10,16,17]. Accordingly, *fus3-3 *is more resistant than WT to thermoinhibition at 32°C. Surprisingly, thermoinhibition of WT immature green seeds is not dependent on ABA biosynthesis, as it cannot be rescued by fluridone. Thus, it appears that different mechanisms may regulate immature and mature seed germination at supraoptimal temperature. In the future, the sensitivity of different stages of immature seeds should be assessed in order to determine the role of ABA in thermoinhibition of immature seeds. Indeed, two peaks of ABA occur during seed development in Arabidopsis and many species; the first maternally-derived ABA peak is required to prevent precocious germination while the second ABA peak is made by the embryo and important to induce and maintain dormancy [2,3]. Thus, the response of immature seeds to fluridone may be dependent on the stage of development.

### Transcriptomic analysis of WT seeds imbibed at supraoptimal temperature

A transcriptomic analysis of seeds imbibed at 32°C shows differential expression of several genes involved in ABA and GA metabolisms as well as signaling. Among the ABA biosynthetic genes, *NCED1, NCED5*, *NCED9*, *ABA1 *and *ABA2 *showed increased expression at 12 and/or 24 h, while *CYP707A2*, the most abundant ABA catabolic gene during germination [39], showed a transient reduction of expression at 12 h. These changes in gene expression are consistent with previous quantifications of transcript levels of several ABA metabolic genes during imbibition at 34°C [39]. The increase in *FUS3 *mRNA level at 12 and 24 h parallels that of the ABA biosynthetic genes. Interestingly, all ABA metabolic genes identified in this microarray with the exception of *NCED1 *contain RY elements, which interact with B3-domain proteins [24-26]. Since *FUS3 *positively regulates ABA levels, these genes may be directly regulated by *FUS3 *and/or other B3 domain proteins.

Genetic analysis indicates that ABA biosynthesis and signaling are required for thermoinhibition of germination and also for the acquisition of thermotolerance during vegetative growth [39,48,53]. Differences were observed in the degree of responses to high temperature of single or higher order ABA biosynthetic mutants, with *aba1 *and the triple *nced2 nced5 nced9 *mutants being strongly resistant to thermoinhibition [39,48]. In agreement, *ABA1*, *NCED5 *and *NCED9 *show the highest increase in transcript level in our microarray (Figure 6).

*ABA insensitive *(*abi*) mutants affected in ABA signaling display different sensitivities to high temperature; for example, *abi1 *is strongly resistant to thermoinhibition, while *abi3 *is moderately resistant and *abi2*, *abi4 *and *abi5 *are sensitive to thermoinhibition [48]. Several ABA signaling components show altered expression during imbibition at high temperature (Figure 6), including several members of the ABA receptor family (*PYLs/RCARs*) and downstream signaling and response genes (*PP2C *phosphatases, *SnRK *kinases and transcription factors) [44,54,55]. The regulation and function of most of these genes during germination at high temperature has not been tested. Together with the fact that not all ABA signaling mutants tested so far are resistant to thermoinhibition, this suggests that different components of the ABA pathway are used to delay or inhibit seed germination at high temperatures.

Our transcriptomic analysis shows that the expression levels of several GA biosynthetic genes were reduced in response to high temperature. Interestingly, *GA3ox1*, *GA3ox2 *and *GA20ox1*, which show the strongest and prolonged reduction in mRNA levels, are under *FUS3*-mediated repression [17,18]. Genetic and chemical studies have shown that thermoinhibition of seed germination in Arabidopsis requires the suppression of GA biosynthesis and signaling [39]. Indeed, thermoinhibition can be partly alleviated by the addition of exogenous GA. Furthermore, loss-of-function mutations in some negative regulators of GA signaling (*spy *and *rgl2*) confer resistance to thermoinhibition, while mutations in several others (*rgl1*, *rgl3*, *rga *and *gai*) do not [39]. Analysis of multiple combinations of DELLA mutants may be required to fully understand the role of GA signaling components in thermoinhibition. Interestingly, our transcriptomic analysis shows that repression of GA signaling at high temperature starts with the transient downregulation of *SLY1 *at 12 h and is followed by the upregulation of *GID1A *and *SPY *at 24 h (Figure 6). Thus, downregulating *SLY1*, which encodes an F-box protein involved in the degradation of multiple DELLA proteins [56,57], may be sufficient to dampen GA signaling during germination at high temperature. The induction at 24 h of *GID1A*, coding for the most abundant GA receptor in dry and imbibed seeds, is possibly due to a feedback regulation triggered by changes in the ABA/GA ratio, as previously suggested [58]. The increase in *GID1A *level at 24 h may also counteract the effect of decreased *SLY1 *mRNA at this time point, since *GID1 *overexpression down-regulates DELLA repression [59]. Genetic analysis of *sly *and *gid *mutants is needed to dissect the role of GA in thermoinhibition of germination.

Seed-specific and seed-maturation related genes are induced by high temperature at 12 and 24 h. A large number of these genes, which are among the highest expressed in dry seeds [30], also show the highest abundance of transcripts in seeds imbibed at 32°C (Additional files 2, 3, 4). A survey of public microarrays shows that most of the *SSP/LEA *genes displaying highest mRNA levels during seed imbibition at 32°C are preferentially expressed during seed development [60]. Furthermore, most of the *SSP/LEA *genes showing increased expression at 32°C, also show induction by ABA in seeds or seedlings [60]. This suggests that at high temperature, the expression levels of *SSP/LEA *genes may fluctuate in response to changes in ABA level. This also corroborates the view that *SSP/LEA *genes are strongly regulated by ABA, but play different roles beyond seed maturation [41]. Among the known global regulators of late embryogenesis, *FUS3 *is the only gene induced by HS in our microarray, and thus may play a predominant role in the regulation of seed-specific programs during germination at high temperature (Additional files 2, 3, 4). This was surprising, as *abi3 *mutants have been recovered in genetic screens for high-temperature resistant mutants [48]. The reduced sensitivity of *abi3 *to high temperature during germination is likely a consequence of the inability of this mutant to respond to ABA. Possibly, high temperature may affect translational and/or posttranslational regulation of ABI3 and/or other components required to act in concert with ABI3, such as ABI5, without affecting *ABI3 *transcription directly [61]. Although *ABI4 *and *5 *expression levels is altered at 32°C, *abi5 *and also *abi4 *mutants have WT responses in thermoinhibition of seed germination [48]. Several uncharacterized *bZIP *genes change in expression level at 12 and/or 24 h after heat stress and may play more predominant roles than *ABI4 *and *ABI5 *during germination at high temperatures (Additional file 3, Additional file 4).

Several stress-related genes are induced by high temperature in our microarrays, including *HSF*, *HSP *as well as *LEA *genes, most of which are abundant during seed maturation and are also thought to play a role in several abiotic stress responses [41,62]. Among the HS-related genes, *HSFA9 *expression in vitro has been shown to be induced by ABI3, but not FUS3. Furthermore, *HSFA9 *mRNA is ~10-fold higher in seeds imbibed on ABA, suggesting a preferential regulation of *HSFA9 *by ABA and ABI3 [31,60]. Although *ABI3 *transcripts do not increase in seeds imbibed at high temperature, activation of the *HSFA9 *promoter during HS may require additional factors beside ABI3. Seed imbibition at high temperature induces the expression of several *HSFs *at different times, some of which (*HSFA2*) play important roles during the acquisition of thermotolerance in seedlings, while others (*HSFA4 *and *HSFC1*) have not been previously shown to be regulated by HS. This is possibly due to the long HS exposure time (12 and 24 h) and stage of development (seed imbibition) used in this microarray compared to previous ones [43,63-65]. The role of these *HSF *in thermoinhibition and during prolonged HS remains to be elucidated.

## Conclusion

In this study, we have uncovered a novel function for the master regulator of seed maturation, *FUS3*, in delaying germination and inhibiting seedling growth at supraoptimal temperature. FUS3 mRNA and protein levels are induced *de novo *when seeds are imbibed at supraoptimal temperature. Furthermore, hyperdormant *ML1:FUS3-GFP *seeds are hypersensitive to thermoinhibition, and this is partly dependent on *de novo *ABA synthesis. Physiologically, this is important since increased dormancy can delay or prevent germination at high temperatures and thus has a protective role for the embryo. Transcriptomic analysis of seeds imbibed at high temperature reveals that a complex program is activated, which involves not only the regulation of heat and dehydration response genes to adjust cellular functions, but also the induction of ABA and repression of GA metabolism and response genes. Finally, our study shows that seeds imbibed at high temperature also activate the expression of genes involved in seed-maturation programs, and that *FUS3 *is the only master regulator of late embryonic development activated during imbibition at supraoptimal temperature. Considering *FUS3 *is induced by heat stress, regulated by ABA/GA levels and itself regulates ABA/GA levels and seed maturation programs, this gene may play an important role in seed responses to high temperature.

We propose a model where FUS3 protein and ABA levels increase during seed imbibition at supraoptimal temperature to delay or inhibit germination through shared as well as independent pathways (Figure 7). Once induced by HS, FUS3 may functions to delay seed germination and prevent seedling growth under unfavorable conditions through positive regulation of ABA synthesis. ABA in turn stabilizes FUS3 levels establishing a positive feedback regulation. FUS3 and ABA negatively regulate GA biosynthesis to delay or prevent germination at supraoptimal temperature.

## Methods

### Plant material, growth conditions and seed germination

For germination assays, 50-100 Arabidopsis WT (Columbia ecotype) or *ML1:FUS3-GFP *(*MFG*; [17]) overexpressing dry seeds were sterilized and imbibed on filter paper placed on MS plates for the indicated times. The loss-of-function *fus3-3 *seeds [10] were processed in the same way, but they were harvested from immature (green/yellow) siliques because of their desiccation intolerance phenotype [10]. All germination assays were conducted in triplicates. Seeds were germinated under constant light in controlled seed incubators at 21 or 32°C. For the recovery period, filter papers were transferred to a fresh MS plate at 21°C for 6 days. Germination rates were scored based on radicle protrusion. Two independent experiments were conducted and a typical result is shown.

### Confocal microscopy

Imbibed *FUS3:GFP *[19] and *FUS3:FUS3-GFP (FFG)*[17] embryos were dissected from the seed coats, mounted on slides in water and imaged by confocal microscopy as previously described [19]. The same confocal settings were used to detect GFP fluorescence intensities at 21 and 32°C.

### Immunoblots

Isolation of proteins and immunoblots were performed as described [19]. GFP and FUS3-GFP were detected with a rabbit anti-GFP (1:3000) polyclonal antibody (Abcam) and Donkey anti-rabbit conjugated to Horse Radish Peroxidase (HRP) (1:10000) antibody (Cederlane).

### Microarray analysis and quantitative real time PCR (qPCR)

Seeds were sterilized and imbibed on filter paper placed on MS plates for the indicated times. Seeds were germinated under constant light in controlled seed incubators at 21 or 32°C and samples were collected after 1, 12 and 24 h. Total RNA extraction and qPCR was done as previously described using primers listed in Additional file 11[19]. The mean value of three replicates was normalized using *ACTIN7 *or *UBC28 *as the internal control. Results are plotted as the ratio to the lowest detected level. Two independent experiments were conducted with similar results and one is shown. RNA was used for cDNA synthesis, labeling, and hybridization of Affymetrix ATH1 arrays according to the Affymetrix GeneChip Expression Analysis technical manual (http://www.affymetrix.com) by the CAGEF Affymetrix GeneChip Facility (Cell & Systems Biology, University of Toronto). Duplicate biological replicates were hybridized to separate arrays. Data file quantification and normalization were performed using the GCOS/MAS5 algorithm with a TGT value of 100. Data quality was confirmed through the use of internal controls and by comparing replicates. Data were then analyzed using a combination of tools including the Eisen lab's Cluster and TreeView programs [66], the Bio-Array Resource at the University of Toronto [67], with BioConductor/R [68] and with Microsoft Excel. An average expression value for each gene for each experimental condition was generated from the normalized data from the duplicate arrays. Genes were labeled as present only if the probability of detection was *P *< 0.05. Data were validated by comparison with real-time PCR for a representative set of genes (Additional file 12). Data were further filtered such that genes were excluded if there was less than a 1.8-fold change (log_2 _0.85). Genes that passed this filtering were analyzed further. Promoter element analysis was performed on 1,000 bp of upstream gene sequences using Promomer (http://bar.utoronto.ca; [67]) and Motif Analisis from TAIR (http://www.arabidopsis.org/tools/bulk/motiffinder/index.jsp). The AgriGO web-based tool (http://bioinfo.cau.edu.cn/agriGO) was used for the Gene Ontology (GO) enrichment analysis [40]. Further expression analysis of selected gene groups was conducted using eNorthern (http://bar.utoronto.ca) [67]. Cluster analysis was done using the Eisen lab's Cluster program [66] on the fold-change data set over three time points. Self-organizing maps were first generated across the genes, and the membership of the maps was used to instruct the gene cluster order during hierarchical clustering using the Pearson (centered) correlation coefficient and average linkage. TreeView was used to visualize the resulting clustergram and data matrix.

## Authors' contributions

SG designed the assays, supervised RSC and funded this study. SG and SRC wrote the manuscript and prepared the figures. RSC performed all the biological experiments, including germination assays, RNA and protein extractions, cDNA synthesis and qRT-PCR, western blot, confocal microscopy (Figures 1,2,3,5,6). HN and NP performed microarray data quality, statistical and cluster analyses (Figure 4, Additional files 2, 3, 4). All authors read and confirmed the manuscript.

## Supplementary Material

Additional file 1 **FUS3-GFP protein expression in *FUS3:FUS3-GFP *seeds during imbibition at 32°C, and in the presence or absence of ABA or paclobutrazol at 21°C**. (A, B) Confocal images showing FUS3-GFP fluorescence in the epidermis of *FUS3:FUS3-GFP *embryos. Seeds were imbibed on MS media with or without hormones or inhibitors for 72 h. During germination on MS media, the FUS3-GFP fluorescence is detected at 32°C (left two panels; red bar), but not at 21°C (black bar) by confocal microscopy. Treatments with either ABA (0.4, 0.8, 1.2 μM) or paclobutrazol (PAC; 0.4 μM) at 21°C are not sufficient to induce FUS3-GFP fluorescence at 72 h after imbibition (HAI). Similar results were obtained at 48 HAI (data not shown). Duplicate experiments were conducted and one is shown. Comparable confocal settings were used in all images. A) Green channel, GFP; B) Merge image of green channel (GFP) and red channel (propidium iodide). Click here for file

Additional file 2 **List of upregulated and downregulated genes at 1 h**. List of upregulated (tab 1) and downregulated (tab 2) *HSR *genes in seeds imbibed at 32°C for 1 h compared to the 21°C control, and top highest expressed or repressed genes at 1 h (tab 3). Genes were filtered as described in the methods. Click here for file

Additional file 3 **List of upregulated and downregulated genes at 12 h**. List of upregulated (tab 1) and downregulated (tab 2) *HSR *genes in seeds imbibed at 32°C for 12 h compared to the 21°C control, and top highest expressed or repressed genes at 12 h (tab 3). Genes were filtered as described in the methods. Click here for file

Additional file 4 **List of upregulated and downregulated genes at 24 h**. List of upregulated (tab 1) and downregulated (tab 2) *HSR *genes in seeds imbibed at 32°C for 24 h compared to the 21°C control, and top highest expressed or repressed genes at 24 h (tab 3). Genes were filtered as described in the methods. Click here for file

Additional file 5 **Summary of GO-terms enrichments in upregulated and downregulated genes**. GO-terms enrichments in upregulated and downregulated genes at each time point using agriGO [40]. The Y-axis is the percentage of genes mapped by the term, and represents the abundance of the GO term. The percentage for each time point is calculated by the number of genes mapped to the GO term divided by the number of all genes in each time point (blue columns). The same calculation was applied to a default reference list (green columns). The X-axis is the GO terms definition. A detailed representation of the sub-biological functions within the GO-term categories can be seen in Additional files 6-10. Click here for file

Additional file 6 **GO-terms enrichments in upregulated genes at 1 h**. Hierarchical tree graph of overrepresented GO terms in biological process categories generated by SEA using AgriGO [40]. Boxes in the graph represent GO terms labeled by their GO ID, term definition and statistical information. The significant term (adjusted *P *≥ 0.05) are marked with color, while non-significant terms are shown as white boxes. In the diagram, the degree of color saturation of a box is positively correlated to the enrichment level of the term (going from yellow, *P *= 0.05 to red, *P *= 5e-10). Solid, dashed, and dotted lines represent two, one and zero enriched terms at both ends connected by the line, respectively. Click here for file

Additional file 7 **GO-terms enrichments in upregulated genes at 12 h**. Click here for file

Additional file 8 **GO-terms enrichments in downregulated genes at 12 h**. Click here for file

Additional file 9 **GO-terms enrichments in upregulated genes at 24 h**. Click here for file

Additional file 10 **GO-terms enrichments in downregulated genes at 24 h**. Click here for file

Additional file 11 **Primers used for qPCR**. Sequences of primers used in qPCR reactions. Click here for file

Additional file 12 **qPCR validation of selected genes differentially regulated in the microarray**. A) Relative expression levels of select *HSR *genes measured by qPCR. *ACTIN7 *was used as the internal control. The mean value of three replicates was normalized using *ACTIN 7 *as the internal control. Results are plotted as the ratio to the lowest detected level. Two independent experiments were conducted with similar results and one is shown. B) Fold change (Log_2_) expression at 32°C versus 21°C of genes shown in A. Click here for file
