# Supplementary figures and images for: The role of the Arabidopsis FUSCA3 transcription factor during inhibition of seed germination at high temperature
Source: BMC Plant Biol. 2012 Jan 27;12:15. doi: 10.1186/1471-2229-12-15 (PMC3296646; doi:10.1186/1471-2229-12-15)

***FUS3:FUS3-GFP (FFG)***  
(72 hours after imbibition)

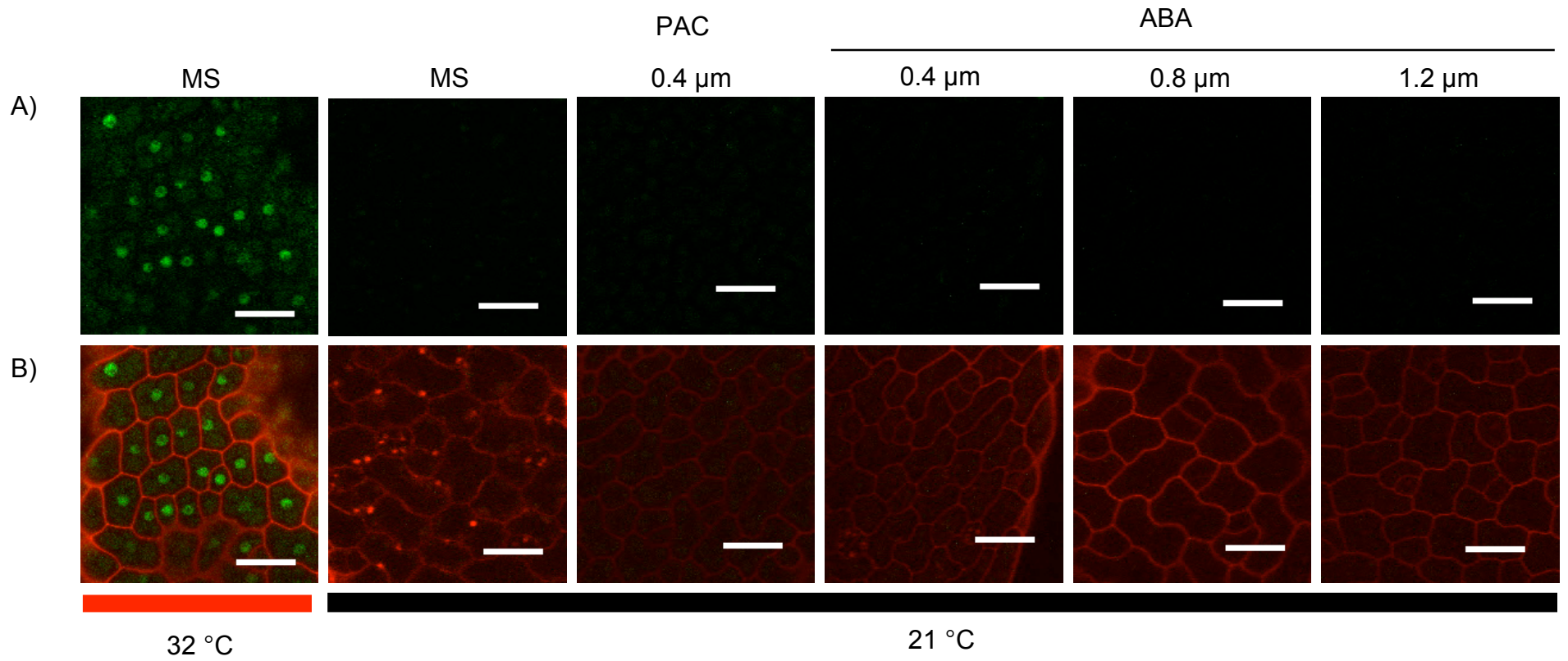

Supplement: Additional file 1 — FUS3-GFP protein expression in FUS3:FUS3-GFP seeds during imbibition at 32°C, and in the presence or absence of ABA or paclobutrazol at 21°C. (A, B) Confocal images showing FUS3-GFP fluorescence in the epidermis of FUS3:FUS3-GFP embryos. Seeds were imbibed on MS media with or without hormones or inhibitors for 72 h. During germination on MS media, the FUS3-GFP fluorescence is detected at 32°C (left two panels; red bar), but not at 21°C (black bar) by confocal microscopy. Treatments with either ABA (0.4, 0.8, 1.2 μM) or paclobutrazol (PAC; 0.4 μM) at 21°C are not sufficient to induce FUS3-GFP fluorescence at 72 h after imbibition (HAI). Similar results were obtained at 48 HAI (data not shown). Duplicate experiments were conducted and one is shown. Comparable confocal settings were used in all images. A) Green channel, GFP; B) Merge image of green channel (GFP) and red channel (propidium iodide). [file 1471-2229-12-15-S1.PDF]

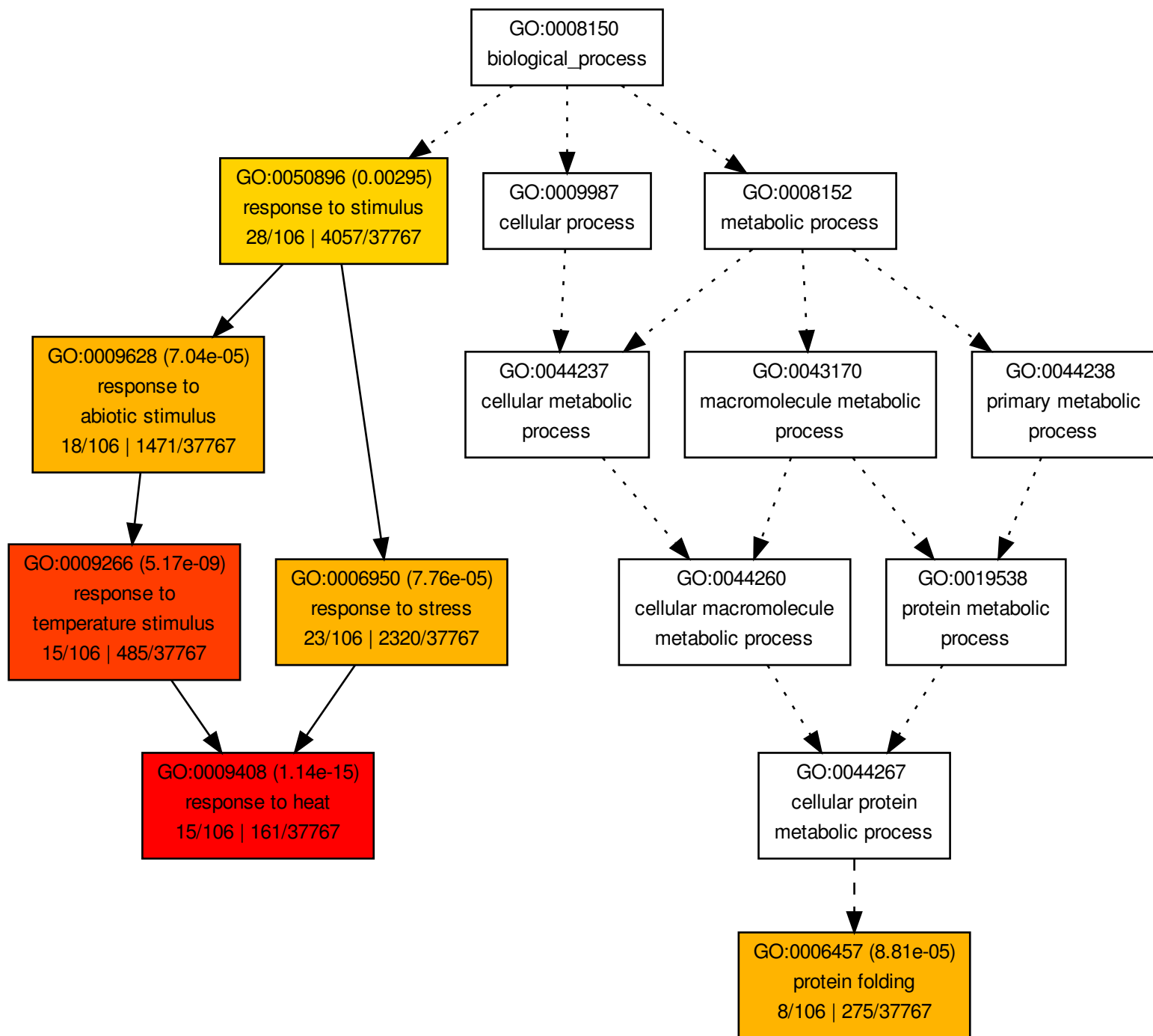

Supplement: Additional file 6 — GO-terms enrichments in upregulated genes at 1 h. Hierarchical tree graph of overrepresented GO terms in biological process categories generated by SEA using AgriGO [40]. Boxes in the graph represent GO terms labeled by their GO ID, term definition and statistical information. The significant term (adjusted P ≥ 0.05) are marked with color, while non-significant terms are shown as white boxes. In the diagram, the degree of color saturation of a box is positively correlated to the enrichment level of the term (going from yellow, P = 0.05 to red, P = 5e-10). Solid, dashed, and dotted lines represent two, one and zero enriched terms at both ends connected by the line, respectively. [file 1471-2229-12-15-S6.PDF]

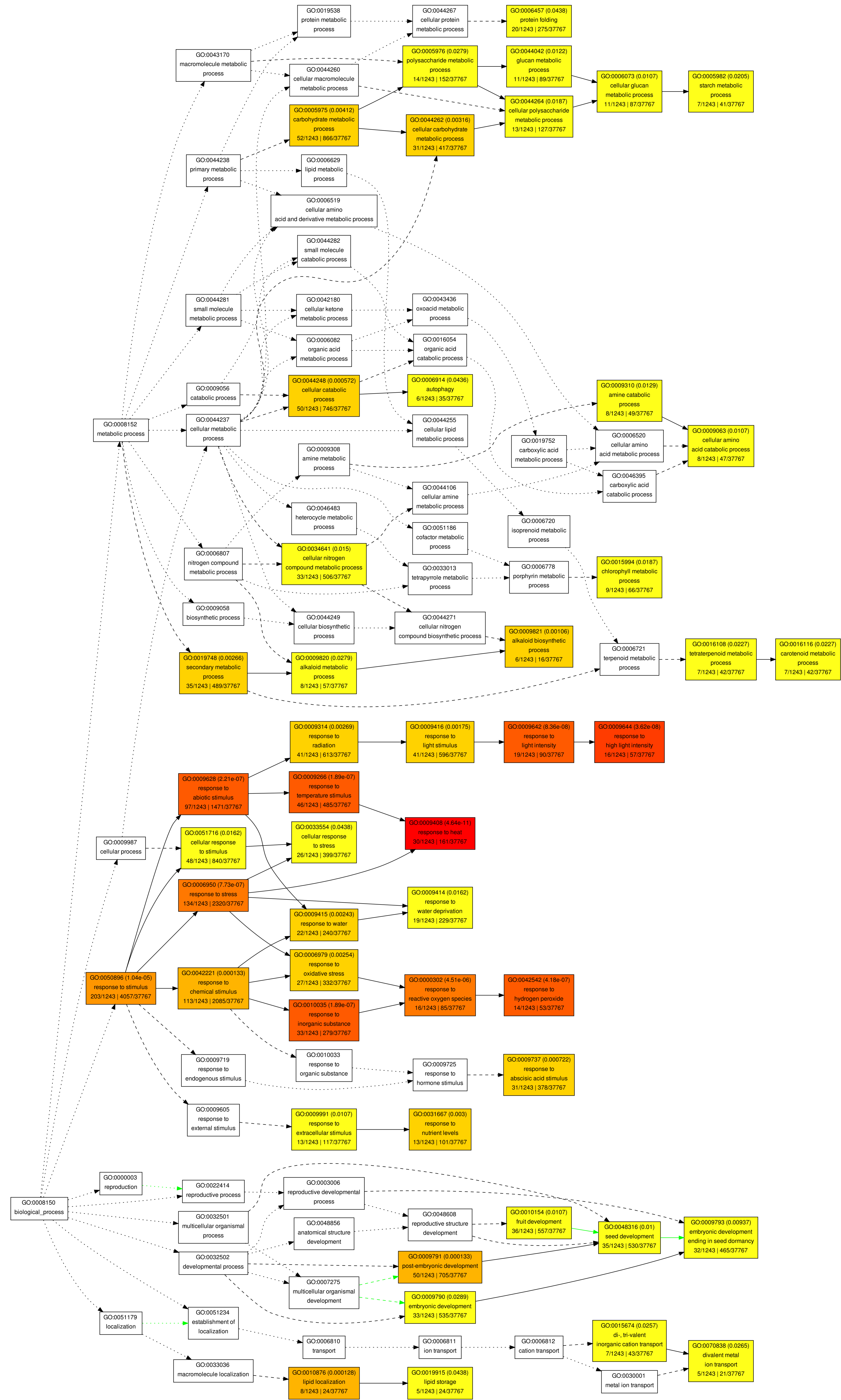

Supplement: Additional file 7 — GO-terms enrichments in upregulated genes at 12 h. [file 1471-2229-12-15-S7.PDF]

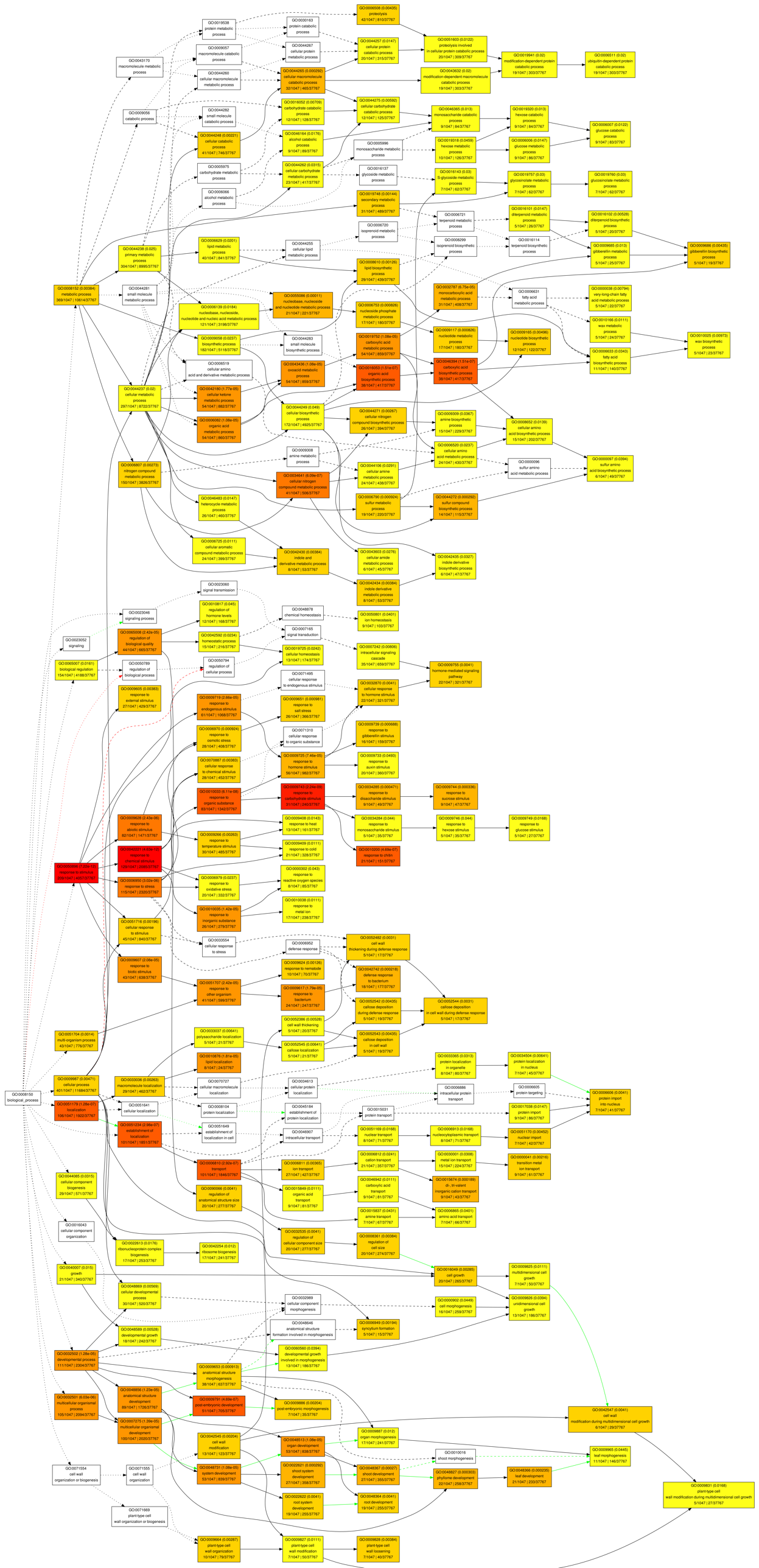

Supplement: Additional file 8 — GO-terms enrichments in downregulated genes at 12 h. [file 1471-2229-12-15-S8.PDF]

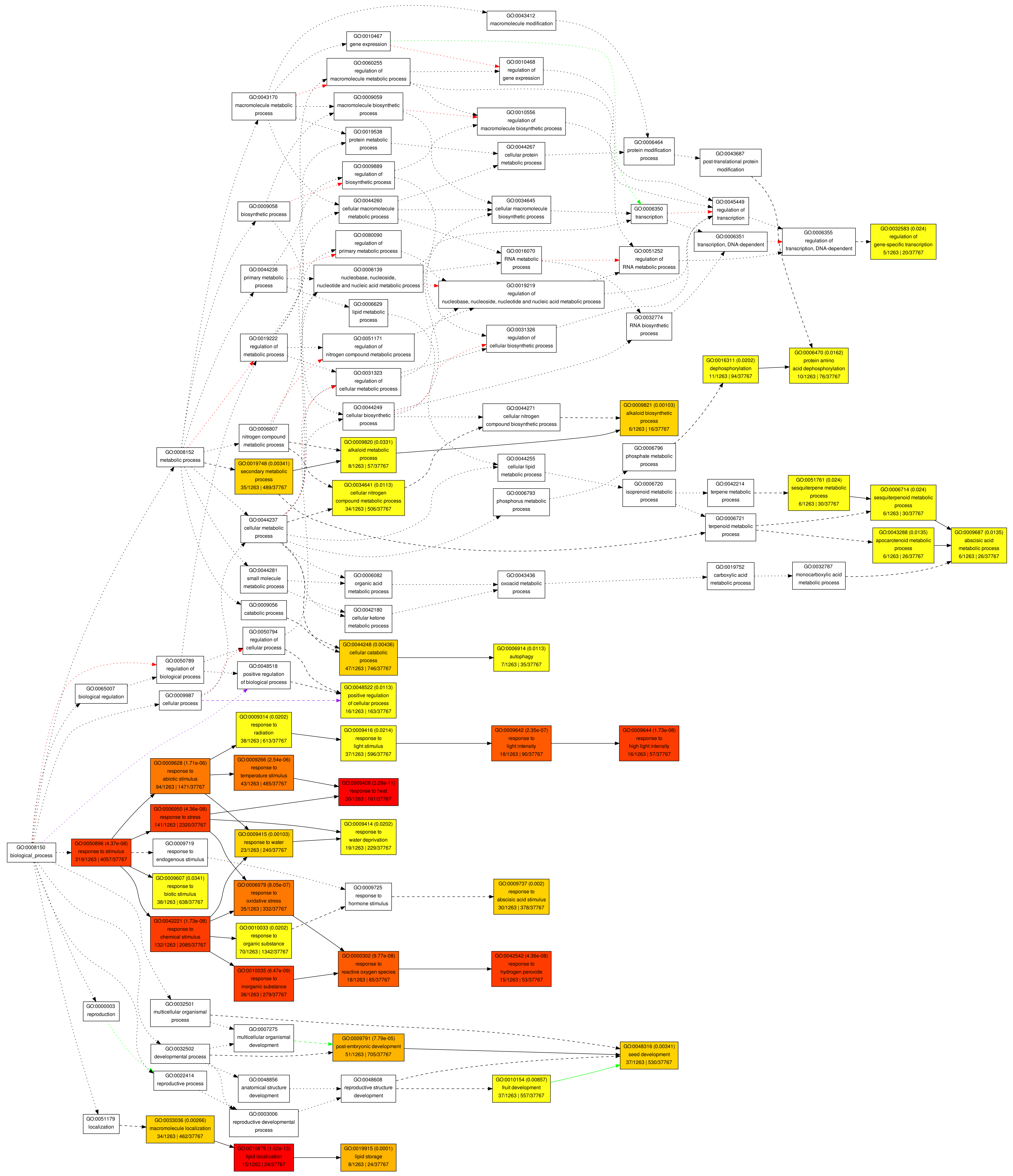

Supplement: Additional file 9 — GO-terms enrichments in upregulated genes at 24 h. [file 1471-2229-12-15-S9.PDF]

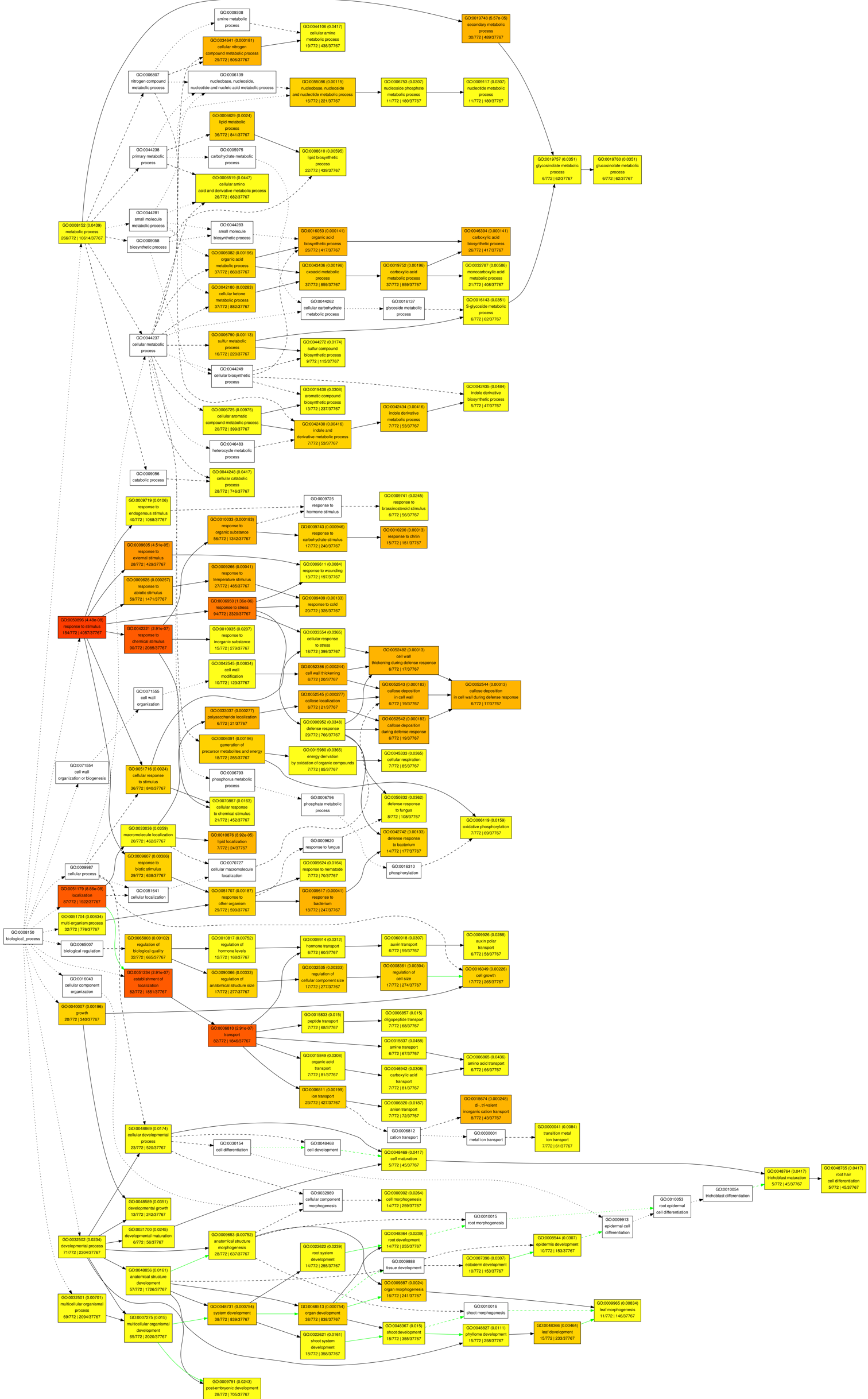

Supplement: Additional file 10 — GO-terms enrichments in downregulated genes at 24 h. [file 1471-2229-12-15-S10.PDF]

**A**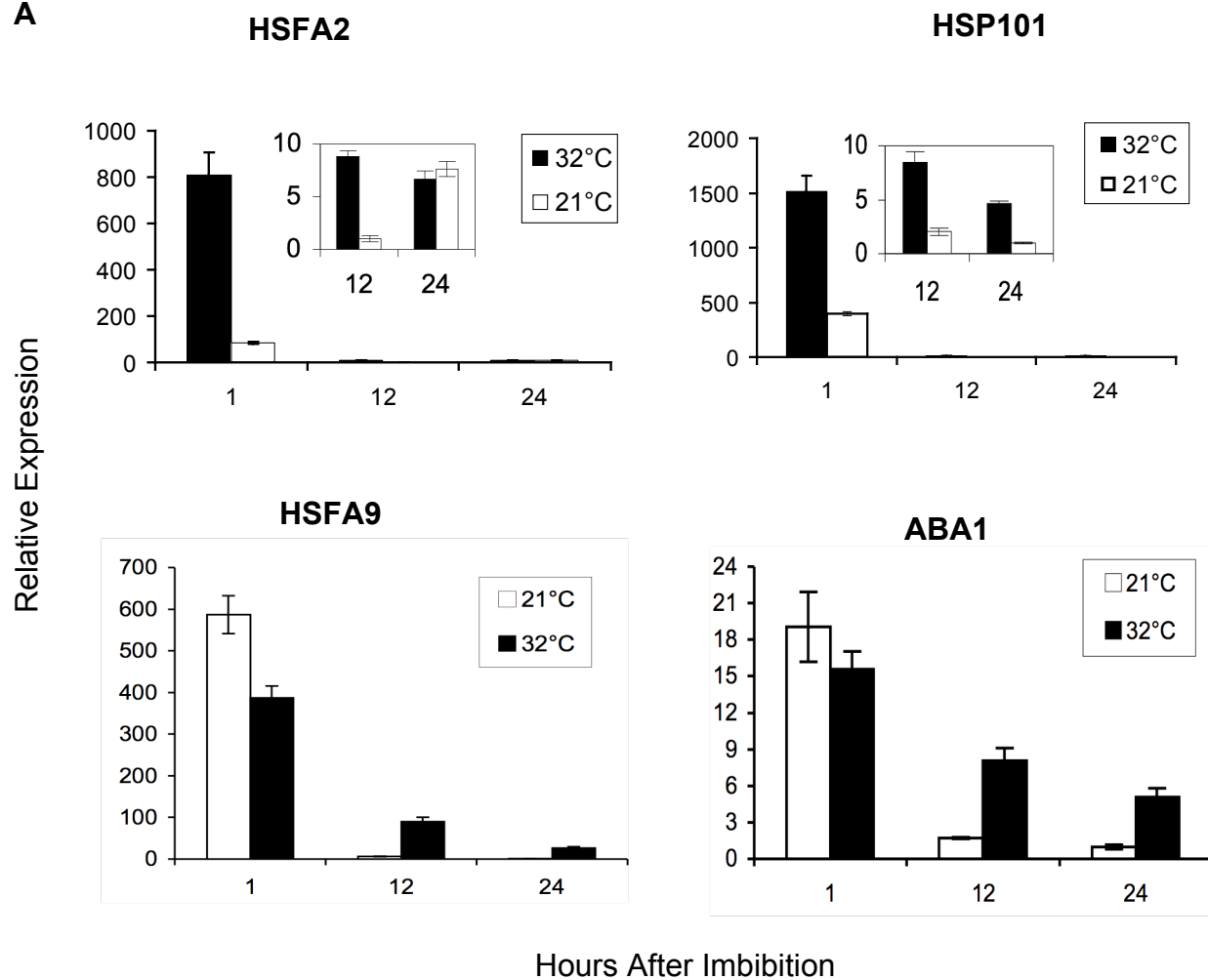**B**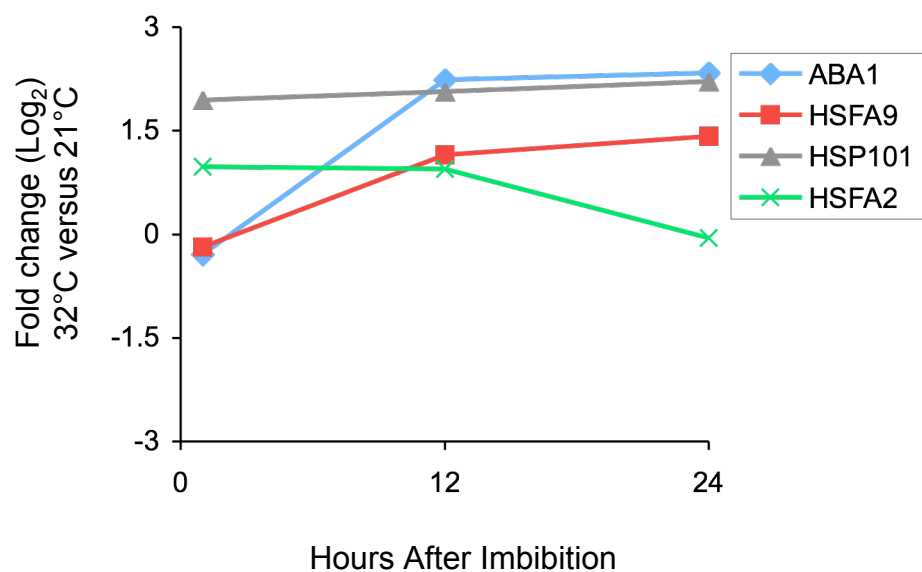

Supplement: Additional file 12 — qPCR validation of selected genes differentially regulated in the microarray. A) Relative expression levels of select HSR genes measured by qPCR. ACTIN7 was used as the internal control. The mean value of three replicates was normalized using ACTIN 7 as the internal control. Results are plotted as the ratio to the lowest detected level. Two independent experiments were conducted with similar results and one is shown. B) Fold change (Log2) expression at 32°C versus 21°C of genes shown in A. [file 1471-2229-12-15-S12.PDF]
