# Supplementary material for: The role of the Arabidopsis FUSCA3 transcription factor during inhibition of seed germination at high temperature
Source: BMC Plant Biol. 2012 Jan 27;12:15. doi: 10.1186/1471-2229-12-15 (PMC3296646; doi:10.1186/1471-2229-12-15)

UPREGULATES *HSRs*

DOWNREGULATED *HSRs*

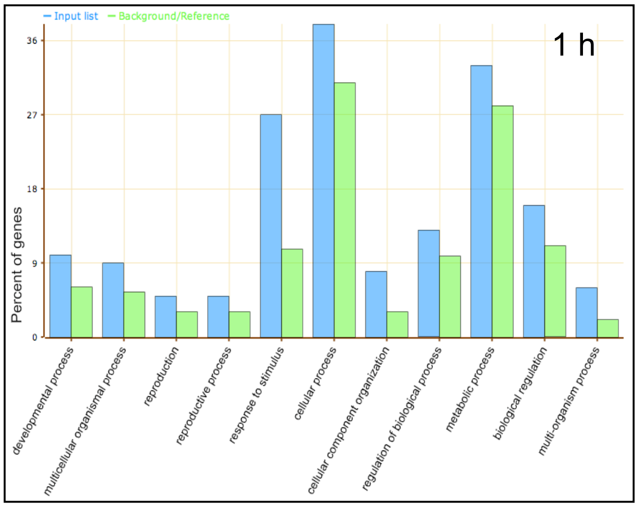

No significant GO term enrichment was found in the list of downregulated genes at 1h

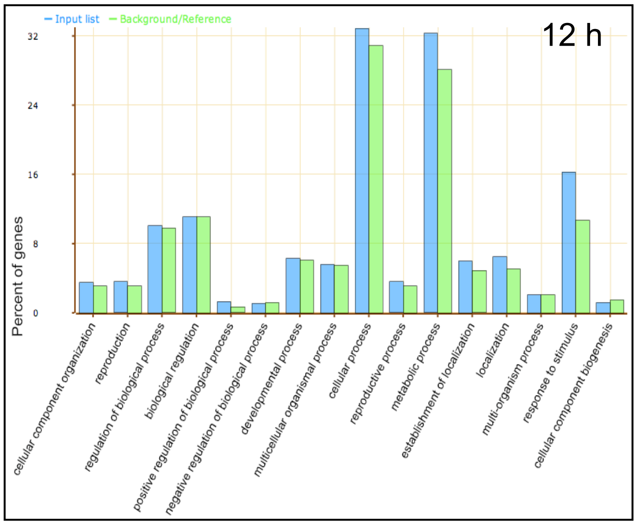

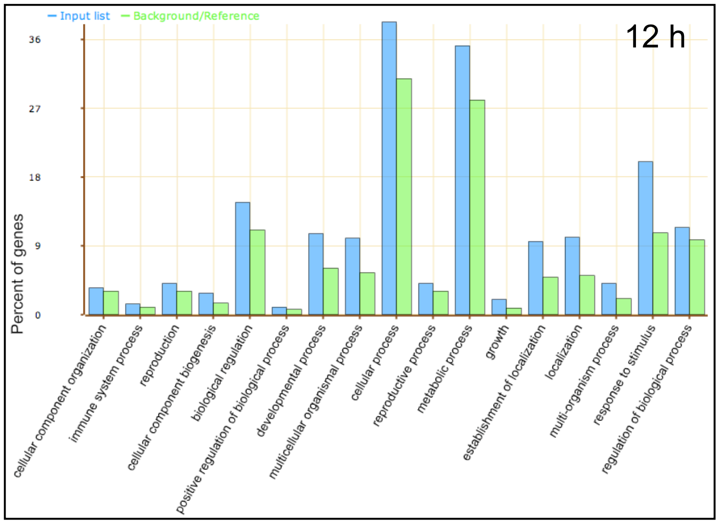

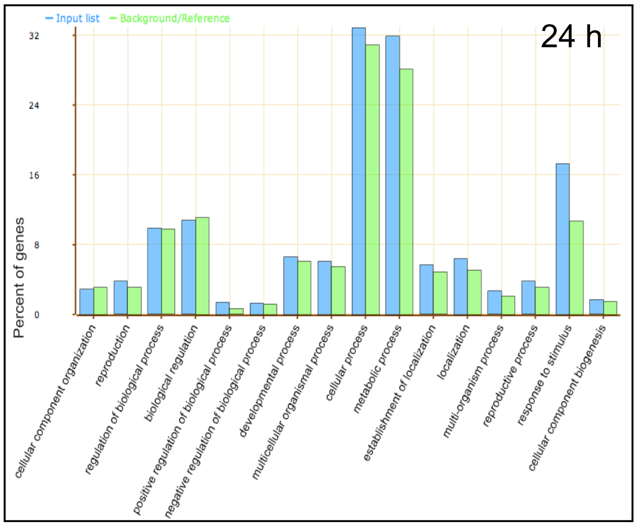

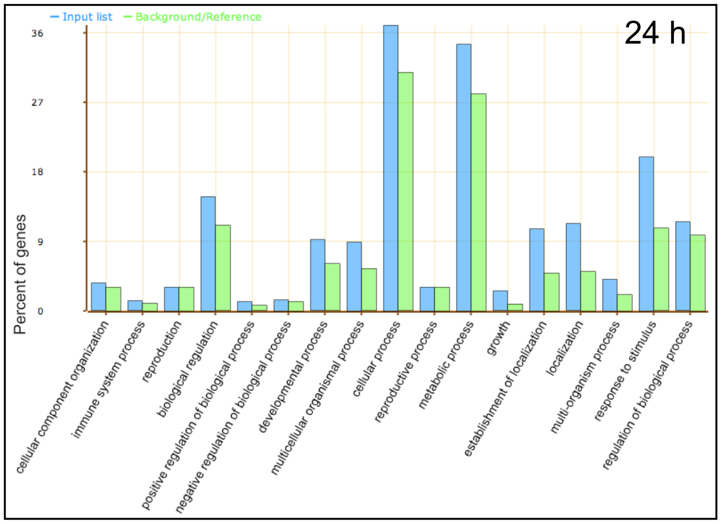

Supplement: Additional file 5 — Summary of GO-terms enrichments in upregulated and downregulated genes. GO-terms enrichments in upregulated and downregulated genes at each time point using agriGO [40]. The Y-axis is the percentage of genes mapped by the term, and represents the abundance of the GO term. The percentage for each time point is calculated by the number of genes mapped to the GO term divided by the number of all genes in each time point (blue columns). The same calculation was applied to a default reference list (green columns). The X-axis is the GO terms definition. A detailed representation of the sub-biological functions within the GO-term categories can be seen in Additional files 6-10. [file 1471-2229-12-15-S5.PDF]
